# Indirect decompression *via* oblique lumbar interbody fusion is sufficient for treatment of lumbar foraminal stenosis

**DOI:** 10.3389/fsurg.2022.911514

**Published:** 2022-08-18

**Authors:** Sheng-Chieh Tseng, Yu-Hsien Lin, Yun-Che Wu, Cheng-Min Shih, Kun-Hui Chen, Cheng-Hung Lee, Chien-Chou Pan

**Affiliations:** ^1^ Department of Orthopedic Surgery, Taichung Veterans General Hospital, Taichung, Taiwan; ^2^ Department of Physical Therapy, HungKuang University, Taichung, Taiwan; ^3^ Department of Nursing, Jenteh Junior College of Medicine, Nursing and Management, Miaoli, Taiwan; ^4^ Department of Computer Science and Information Engineering, College of Computing and Informatics, Providence University, Taichung, Taiwan; ^5^ Department of Biomedical Engineering, College of Intelligent Technology, HungKuang University, Taichung, Taiwan; ^6^ College of Medicine, National Chung Hsing University, Taichung, Taiwan; ^7^ Department of Food Science and Technology, Hungkuang University, Taichung, Taiwan; ^8^ Department of Rehabilitation Science, Jenteh Junior College of Medicine, Nursing and Management, Miaoli, Taiwan

**Keywords:** oblique lumbar interbody fusion, lumbar foraminal stenosis, indirect decompression, direct decompression, laminotomy, laminectomy

## Abstract

Oblique lumbar interbody fusion (OLIF) is a popular technique for the treatment of degenerative lumbar spinal disease. There are no clear guidelines on whether direct posterior decompression (PD) is necessary after OLIF. The purpose of this study was to analyze the effect of the indirect decompression obtained from OLIF in patients with lumbar foraminal stenosis. We retrospectively reviewed 33 patients who underwent OLIF surgery for degenerative lumbar spinal disease between 1 January 2018, and 30 June 2019. The inclusion criteria included patients who were diagnosed with lumbar foraminal stenosis by preoperative MRI. The exclusion criteria included the presence of central canal stenosis, spinal infection, vertebral fractures, and spinal malignancies. The clinical results, evaluated using the visual analogue scale of back pain (VAS-Back), VAS of leg pain (VAS-Leg), and Oswestry disability index (ODI), were recorded. The radiologic parameters were also measured. The VAS-Back, VAS-Leg, and ODI showed significant improvement in both the PD and non-posterior decompression (Non-PD) groups postoperatively (all, *p* < 0.05). Patients in the Non-PD group showed better results than those in the PD group in the VAS-Back at 12- and 24 months postoperatively (0.00 vs. 3.00 postoperatively at 12 months, *p* = 0.030; 0.00 vs. 4.00 postoperatively at 24 months, *p* = 0.009). In addition, the ODI at 24 months postoperatively showed better improvement in the Non-PD group (8.89 vs. 24.44, *p* = 0.038). The disc height in both the PD and the Non-PD groups increased significantly postoperatively (all, *p* < 0.05), but the restoration of foraminal height was significantly different only in the Non-PD group. There was no statistically significant difference in cage position, cage subsidence, fusion grade, or screw loosening between the PD and the Non-PD groups. Indirect decompression *via* OLIF for lumbar foraminal stenosis showed favorable outcomes. The use of interbody cages and posterior instrumentation was sufficient for relieving symptoms in patients with lumbar foraminal stenosis. Additional direct posterior decompression may deteriorate results in the follow-up period.

## Introduction

Spinal fusion is a popular surgical treatment for degenerative lumbar spinal disease such as spinal stenosis, spondylolisthesis, or disc herniation (1). There are various lumbar spinal fusion techniques, including anterior lumbar interbody fusion, posterior lumbar interbody fusion (PLIF), and transforaminal lumbar interbody fusion (TLIF). The retroperitoneal approach, which was first introduced by Mayer in 1997, is a minimally invasive technique for decreasing surgery-related comorbidities (2). Several modifications of this technique were developed in subsequent years. Silvestre et al. used a similar approach, which is referred to as oblique lumbar interbody fusion (OLIF), and presented the first results about complications and morbidities (3). OLIF has the advantages of less blood loss, shorter hospital stays, and faster recovery when compared with conventional posterior approaches (4). Previous studies have confirmed its achievement of indirect neural decompression through the restoration of disc height and extension of the thecal sac (5). Shimizu et al. demonstrated that OLIF had good short-term clinical outcomes, comparable to those obtained with TLIF and PLIF, for severe degenerative lumbar stenosis (6). Some authors have stated that indirect decompression could achieve adequate neural decompression through direct lateral interbody fusion (DLIF), lateral lumbar interbody fusion (LLIF), or extreme lateral interbody fusion (XLIF) (7–9). The fundamental concept of these lateral interbody fusion techniques is the “indirect decompression” effect through the restoration of intervertebral disc height and foraminal height (FH). Shimizu et al. confirmed that lateral interbody fusion without posterior decompression (PD) achieved expansion of the thecal sac and restoration of disc height in severe canal stenosis (7). However, most of these studies have focused on DLIF, LLIF, and XLIF and it remains unclear whether indirect decompression alone is sufficient to relieve low back pain or radicular pain in patients receiving OLIF. Furthermore, 0%–60% of patients who received these indirect decompression procedures underwent additional posterior laminectomy (10, 11). The posterior decompression procedures have the advantage of the direct decompression of the nerve root. However, they may cause iatrogenic injuries to the paravertebral muscles and disruption of the posterior tension mechanism (12). There are no clear guidelines on whether direct posterior decompression is necessary after OLIF.

Therefore, the aim of this study was to analyze the effect of the indirect decompression obtained from OLIF in patients with lumbar foraminal stenosis (FS), in terms of clinical and radiologic outcomes. We also investigated whether additional direct posterior decompression affected the outcomes in these patients.

## Methods

We retrospectively reviewed 33 patients who underwent OLIF surgery for degenerative lumbar spinal disease between 1 January 2018 and 30 June 2019. The inclusion criteria included patients who were diagnosed with lumbar FS by preoperative MRI. The radiologic criteria of lumbar spinal stenosis were summarized in a systematic review article (13). FS is diagnosed by nerve root compression in the foraminal zone with obliteration of the perineural intraforaminal fat (14). The exclusion criteria included the presence of central canal stenosis, spinal infection, vertebral fractures, and spinal malignancies. These patients were divided into posterior decompression (PD) or non-posterior decompression (Non-PD) groups according to whether direct posterior decompression was performed. The minimum follow-up period was at least 24 months. All the surgeries were performed by experienced spine surgeons at our institute.

**Figure 1:**
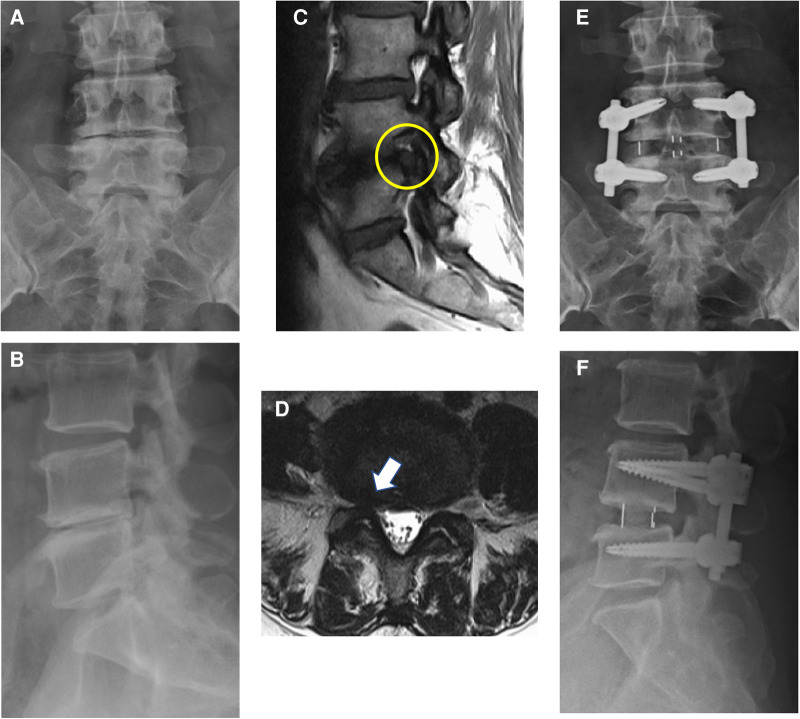
Anterior–posterior view (**A**) and lateral view (**B**) of a 67-year-old woman with degenerative disc disease. Sagittal T1-weighted MRI (**C**) and axial T2-weighted MRI (**D**) showed foraminal stenosis of L4/L5 (yellow circle and white arrow). Postoperative anterior–posterior view (**E**) and lateral view (**F**) of this patient. OLIF, L4/L5, with posterior instrumentation was done.

We performed the OLIF procedure as described by Woods et al. (15). The Clydesdale cage (Medtronic, TN, USA) was used, and a morselized bone allograft or synthetic bone graft substitute (Actifuse, Baxter, IL, USA) was packed into the cage to enable fusion. After the performance of the OLIF procedures, posterior instrumentation with pedicle screws was used in all cases. Importantly, the surgeons informed patients of the pros and cons of additional posterior decompression procedure before the operation. Proper suggestions were provided by the surgeons, and the patients made the final decision on whether the posterior decompression procedure was performed. If direct posterior decompression was planned, the surgeons decided the exact procedure, including laminectomy, laminotomy, or discectomy according to their experience and preference. All the posterior decompression procedures were performed before the insertion of pedicle screws. Adequate decompression of the dural sac and nerve roots at lateral recess and neuroforamen was checked meticulously, and hemostasis was performed (Figure 1).

**Figure 2:**
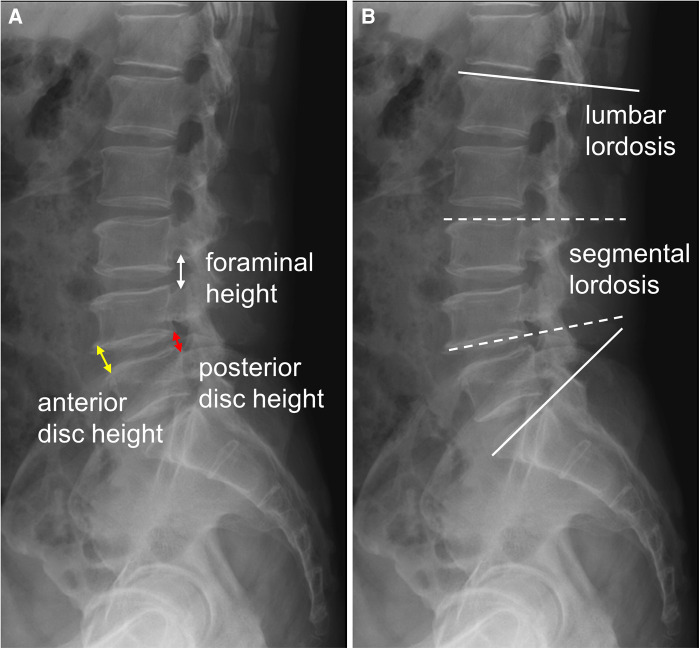
The radiologic parameters (**A**) anterior disc height (ADH, yellow arrow): the distance of the anterior disc space; posterior disc height (PDH, red arrow): the distance of the posterior disc space; average disc height (DH): (ADH + PDH)/2; foraminal height (FH, white arrow): the distance between the pedicles of upper and lower levels; (**B**) lumbar lordosis (LL, solid line): the angle of L1 to S1 upper endplate; segmental lordosis (SL, dotted line): the angle of the upper endplate of the upper vertebra and the lower endplate of the lower vertebra of the index level.

The clinical outcomes were evaluated using the visual analogue scale of back pain (VAS-Back), VAS of leg pain (VAS-Leg), and Oswestry disability index (ODI), which were recorded preoperatively and at the postoperative 1-, 3-, 6-, 12-,and 24-month follow-ups. The minimum clinically important difference (MCID) for the patient-reported outcome measures in this study was defined as a 30% reduction from baseline of pain and disabilities (16). The radiologic parameters, including the index level of the anterior disc height (ADH), posterior disc height (PDH), average disc height (DH), FH, lumbar lordosis (LL), and segmental lordosis (SL), were measured preoperatively, postoperatively, and at the last follow-up in the outpatient clinic (Figure 2). Additionally, we analyzed the cage position and cage-related parameters at the last follow-up time. The normalized mean cage center position was defined as the value of the distance between the cage center to the posterior vertebral border divided by the width of inferior end plate on the lateral view of the x-ray (17). The grading of cage subsidence was determined according to Marchi et al. (18): Grade 0, 0%–24%; Grade I, 25%–49%; Grade II, 50%–74%; and Grade III, 75%–100% collapse of the vertebral end plate. The fusion grade was classified according to Ailon et al. (19): Grade I, definite union; Grade II, probable union; Grade III, probable non-union; Grade IV, definite non-union. Finally, the perioperative parameters and postoperative complications were recorded by chart review.

### Statistical analyses

Statistical analyses were performed using the Statistical Package for the Social Sciences (IBM SPSS version 22.0; International Business Machines Corp., New York, USA). The Friedman test was used for comparison of the postoperative values of each clinical and radiologic outcome with the preoperative values. The Bonferroni test was used for the *post hoc* analysis. The chi-square test was used to compare the qualitative variables between the groups, and the Mann–Whitney *U* test to compare the quantitative variables between the groups. A *p*-value < 0.05 was statistically signiﬁcant.

## Results

A total of 33 patients were included in this study, with 16 patients in the Non-PD group and 17 patients in the PD group. There were no significant differences between the two groups in terms of age, sex, BMI, operative levels, and follow-up time. The patient demographics are given in Table 1.

**Table 1:** The patient demographics.

	Non-PD (*n *=* *16)	PD (*n *=* *17)	*p*-Value
Age (years), median (Q1–Q3)	64.0 (53.5–70.5)	64.0 (56.5–72.0)	0.857
Gender, *n* (%)			0.225
Female	14 (87.5)	11 (64.7)	
Male	2 (12.5)	6 (35.3)	
BMI (kg/m^2^), median (Q1–Q3)	24.8 (22.9–26.6)	23.9 (22.7–26.8)	0.471
Level, *n* (%)			0.460
Single level	10 (62.5)	12 (70.6)	
Two levels	6 (37.5)	4 (23.5)	
Three levels	0 (0.0)	1 (5.9)	
Follow-up time, months, median (Q1–Q3)	33.0 (26.7–35.2)	34.8 (29.5–37.2)	0.428

Non-PD, non-posterior decompression; PD, posterior decompression; BMI, body mass index. The chi-square test was used to compare the qualitative variables between the groups. The Mann–Whitney U test was used to compare the quantitative variables between the groups.

The VAS-Back, VAS-Leg, and ODI showed significant improvement in both the PD and Non-PD groups postoperatively (all, *p* < 0.05) (Table 2). All the pain scales achieved MCID (a reduction of 2.4 points for the VAS of the back and leg) postoperatively at the 1-month follow-up. The disability scores achieved MCID (a reduction of 16.67 points for ODI) at 3 months postoperatively in the Non-PD group and at 6 months postoperatively in the PD group.

**Table 2:** Comparison of the clinical outcomes between the Non-PD and PD groups.

	Non-PD (*n *=* *16) median (Q1–Q3)	PD (*n *=* *17) median (Q1–Q3)	*p*-Value
VAS-Back			
Preop	7.50 (6.25–8.00)	8.00 (7.00–8.00)	0.384
Postop-1M	3.50 (2.25–5.00)	4.00 (2.25–5.75)	0.593
Postop-3M	2.50 (2.00–5.00)	3.00 (2.00–4.75)^*^	0.703
Postop-6M	1.00 (0.00–3.00)^*^	3.00 (2.00–4.75)^*^	0.105
Postop-12M	0.00 (0.00–2.00)^*^	3.00 (1.25–4.75)^*^	0.030^†^
Postop-24M	0.00 (0.00–2.25)^*^	4.00 (2.00–6.00)^*^	0.009^†^
VAS-Leg			
Preop	6.50 (1.25–8.00)	8.00 (6.00–8.50)	0.282
Postop-1M	0.00 (0.00–0.00)^*^	0.00 (0.00–3.75)^*^	0.096
Postop-3M	0.00 (0.00–0.00)^*^	0.00 (0.00–2.75)^*^	0.583
Postop-6M	0.00 (0.00–0.00)^*^	0.00 (0.00–1.50)^*^	0.796
Postop-12M	0.00 (0.00–0.00)^*^	0.00 (0.00–0.00)^*^	0.728
Postop-24M	0.00 (0.00–0.00)^*^	0.00 (0.00–0.00)^*^	0.141
ODI			
Preop	55.56 (42.22–63.89)	55.56 (44.44–65.56)	0.538
Postop-1M	43.33 (38.89–56.67)	46.67 (44.44–55.56)	0.238
Postop-3M	32.23 (22.22–37.78)	42.22 (32.22–48.34)^*^	0.123
Postop-6M	22.23 (11.11–28.89)^*^	37.78 (22.22–48.34)^*^	0.094
Postop-12M	11.11 (6.67–20.00)^*^	32.23 (11.67–48.34)^*^	0.162
Postop-24M	8.89 (8.34–16.11)^*^	24.44 (8.89–46.67)^*^	0.038^†^

Non-PD, non-posterior decompression; PD, posterior decompression; VAS, visual analogue scale; ODI, Oswestry disability index; Preop, preoperative; Postop, postoperative; M, month. Intragroup difference compared with Preop: the Friedman test, the Bonferroni test (the post-hoc analysis). Intergroup difference: the Mann–Whitney U test.

^*^ *p* < 0.05.

^†^ *p* < 0.05.

**Figure 3:**
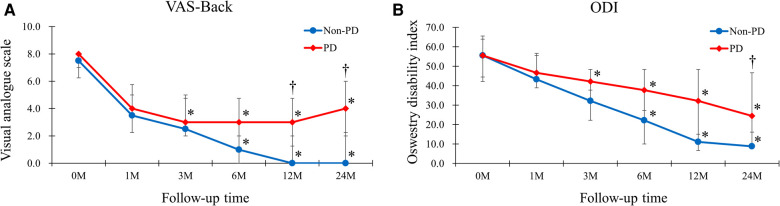
The VAS-Back and ODI of the Non-PD and PD groups. (**A**) Patients in the Non-PD group had better results than those of PD group in the VAS-Back at 12-months and 24-months postoperatively (0.00 vs. 3.00 postoperatively at 12-months, *p* = 0.030; 0.00 vs. 4.00 postoperatively at 24-months, *p* = 0.009). (**B**) The ODI at 24-months postoperatively showed better improvement in the Non-PD group (8.89 vs. 24.44, *p* = 0.038).

Patients in the Non-PD group showed better results than those in the PD group in the VAS-Back at 12 months and 24 months postoperatively (0.00 vs. 3.00 postoperatively at 12 months, *p* = 0.030; 0.00 vs. 4.00 postoperatively at 24 months, *p* = 0.009) (Figure 3). In addition, the ODI at 24 months postoperatively showed better improvement in the Non-PD group (8.89 vs. 24.44, *p* = 0.038).

The ADH, PDH, and DH in both the PD and Non-PD groups increased significantly postoperatively (all, *p* < 0.05) (Table 3). The results were obtained at the last follow-up. The restoration of FH was significantly different only in the Non-PD group. However, the LL and SL had no significant increase after OLIF in both groups.

**Table 3:** Comparison of the radiologic outcomes between the Non-PD and the PD groups.

	Non-PD (*n *=* *22) median (Q1, Q3)	PD (*n *=* *23) median (Q1, Q3)	*p*-Value
ADH (mm)			
Preop	6.80 (5.10, 10.45)	10.45 (7.45, 12.30)	
Postop−Preop	4.10 (2.20, 5.60)^*^	3.35 (2.10, 4.70)^*^	0.318
Last−Preop	5.45 (3.15, 7.80)^*^	3.30 (0.15, 5.15)^*^	0.019^†^
PDH (mm)			
Preop	5.40 (4.50, 5.70)	6.80 (5.40, 7.70)	
Postop−Preop	3.70 (2.90, 3.90)^*^	1.95 (0.95, 3.20)^*^	0.008^†^
Last−Preop	3.40 (3.00, 5.55)^*^	1.80 (0.80, 3.10)^*^	0.001^†^
DH (mm)			
Preop	6.00 (5.10, 7.85)	9.08 (7.00, 10.00)	
Postop−Preop	3.70 (2.95, 5.00)^*^	2.85 (1.75, 4.05)^*^	0.048^†^
Last−Preop	4.60 (3.20, 5.70)^*^	2.60 (0.60, 3.93)^*^	0.002^†^
FH (mm)			
Preop	16.70 (13.10, 18.80)	19.20 (16.45, 21.65)	
Postop−Preop	2.90 (−2.10, 6.40)^*^	1.60 (−0.10, 2.75)	0.453
Last−Preop	1.00 (−1.20, 3.60)	0.30 (−1.75, 3.10)	0.317
LL (degree)			
Preop	35.95 (27.98, 42.98)	31.65 (23.63, 48.45)	
Postop−Preop	3.45 (−3.28, 8.25)	1.50 (−7.25, 9.70)	0.801
Last−Preop	3.40 (−3.23, 12.43)	2.20 (−5.28, 13.43)	0.943
SL (degree)			
Preop	11.20 (4.86, 8.30)	12.60 (7.18, 22.85)	
Postop−Preop	2.25 (−0.85, 4.26)	2.20 (−0.50, 5.58)	0.885
Last−Preop	0.80 (−3.39, 5.73)	2.30 (−2.23, 4.98)	0.857

Non-PD, non-posterior decompression; PD, posterior decompression; ADH, anterior disc height; PDH, posterior disc height; DH, average disc height; FH, foraminal height; LL, lumbar lordosis; SL, segmental lordosis; Preop, preoperative; Postop, postoperative; Last, last follow-up. Intragroup difference: the Friedman test, the Bonferroni test (the post-hoc analysis). Intergroup difference: the Mann–Whitney U test.

^*^ *p* < 0.05.

^†^ *p* < 0.05.

A comparison of the PD and Non-PD groups showed that the latter had a better improvement ratio in terms of ADH, PDH, and DH than the former. There was no significant difference in FH, LL, and SL between the two groups.

There was no statistically significant difference in cage position, cage subsidence, fusion grade, or screw loosening between the PD and the Non-PD groups (Table 4). High-grade cage subsidence (Grades II and III) occurred in 18.2% patients of the Non-PD group and 13% patients of the PD group. Importantly, all the patients achieved adequate spinal fusion on image presentation at the last follow-up time.

**Table 4:** Cage position and cage-related parameters.

	Non-PD (*n *=* *22)	PD (*n *=* *23)	*p-*Value
Normalized mean cage center position, median (Q1–Q3)	0.58 (0.51–0.65)	0.57 (0.55–0.62)	0.982
Cage subsidence, *n* (%)			0.446
Grade 0	8 (36.4)	12 (52.2)	
Grade I	10 (45.5)	8 (34.8)	
Grade II	4 (18.2)	2 (8.7)	
Grade III	0 (0)	1 (4.3)	
Fusion grade, *n* (%)			1.000
Grade I	18 (81.8)	19 (82.6)	
Grade II	4 (18.2)	4 (17.4)	
Grade III	0 (0)	0 (0)	
Grade IV	0 (0)	0 (0)	
Screw loosening, *n* (%)	4 (18.2)	3 (13.0)	0.688

Non-PD, non-posterior decompression; PD, posterior decompression. The chi-square test was used to compare the qualitative variables between the groups. The Mann–Whitney U test was used to compare the quantitative variables between the groups.

The estimated blood loss was similar in both groups (Table 5). Regarding postoperative minor complications, only one patient in each group experienced postoperative ileus. Numbness of the thigh occurred in three patients (18.8%) in the Non-PD group and two patients (11.8%) in the PD group. Besides, in the PD group, one patient had dural tear and another one had superficial wound infection. No major complication or reoperation was recorded in either group.

**Table 5:** Perioperative parameters and postoperative complications.

	Non-PD (*n *=* *16)	PD (*n *=* *17)	*p-*Value
Estimated blood loss (ml), median (Q1–Q3)	300 (212.50–475.00)	320 (225.00–475.00)	0.800
Complications, *n* (%)			
Postoperative ileus	1 (6.3)	1 (5.9)	1.000
Numbness of thigh	3 (18.8)	2 (11.8)	0.656
Delirium	1 (6.3)	0 (0)	0.485
Dural tear	0 (0)	1 (5.9)	1.000
Superficial wound infection	0 (0)	1 (5.9)	1.000

Non-PD, non-posterior decompression; PD, posterior decompression. The chi-square test was used to compare the qualitative variables between the groups. The Mann–Whitney U test was used to compare the quantitative variables between the groups.

## Discussion

Lumbar interbody fusion techniques such as TLIF and PLIF have become well-developed methods for treating degenerative lumbar spinal disease (1). These posterior approaches could decompress the neural elements directly and provide initial stability through the use of interbody cages and pedicle screws. Nevertheless, the posterior structures would be damaged simultaneously (12). OLIF is a lateral-approach technique using the corridor between the psoas muscle and the aorta. It avoids violations of the psoas and lumbosacral plexus injuries and has a high fusion rate (15). It has been shown to significantly improve clinical outcomes, and its fusion rate was 97.9% at 6 months (15). Our data showed a comparatively high fusion rate, with all patients having achieved successful fusion at the 2-year follow-up period. Another study showed that stand-alone minimally invasive lateral interbody fusion could relieve neurologic symptoms and improve the quality of life in selected patient populations (20). Furthermore, the rate of high-grade cage subsidence was 9% and not related directly to the clinical outcomes (20). According to our results, 15% of patients exhibited high-grade cage subsidence. The phenomenon of subsidence was multifactorial, including bone mineral density, disc height, and cage position (21). There was no difference in the ratio of cage subsidence between the Non-PD and the PD groups. Additional posterior decompression procedure may not affect the probability of cage subsidence. Also, the cage was inserted a little anterior to the center of the lower end plate in both groups without intergroup difference in cage position. Yao et al. considered that anterior placement of the TLIF cage may reduce the risk of cage subsidence (21). A systematic review reported that the cage position had no influence on the indirect decompression effect in XLIF (22). More evidence is needed to confirm the relationship between cage position and indirect decompression effect.

Recently, some studies demonstrated that the “indirect decompression” effect *via* OLIF showed good short-term clinical and radiologic outcomes (5, 23). Kim et al. found that OLIF increased the DH and sagittal angle significantly at the 1-year follow-up. However, the FH did not change (24). Shimizu et al. demonstrated similar clinical outcomes between OLIF and conventional TLIF/PLIF in the treatment of severe spinal stenosis, while OLIF was shown to have better radiographic outcomes (6). For adjacent segment disease after posterior lumbar fusion, OLIF has better short-term clinical outcomes and DH restoration than PLIF (4).

In our study, the neurologic symptoms caused by foraminal stenosis were much improved after OLIF. This means the radicular pain caused by nerve root compression at neuroforamen was efficiently relieved by means of the “indirect decompression” effect obtained from OLIF. In addition, the Non-PD patients showed better clinical results than those in the PD group in the VAS score for back pain at 12- and 24 months postoperatively and ODI at 24 months postoperatively. Theoretically, direct posterior decompression such as laminectomy or laminotomy could decompress the neural elements directly. The osteophytes and redundant ligamentum flavum can be removed meticulously, and the nerve root can be released. However, some authors have found that a posterior decompression procedure may cause iatrogenic injuries to the paraspinal musculature and disruptions of the posterior bony structure (12, 25). These additional procedures may contribute to paraspinal muscle atrophy and compromise the result of the index procedure (26). Besides, the integrity of the posterior complex between the fused segments and the adjacent segments could be damaged in laminectomy or laminotomy after lumbar spinal fusion (27). The development of adjacent instability would deteriorate the outcomes of spinal fusion and may lead to adjacent segment disease in the future. This is the reason why the patients in the PD group still had back pain in the 24-month follow-up period. In our opinion, the efficacy of indirect decompression is sufficient for lumbar FS. Additional direct posterior decompression is not necessary in these cases.

On the other hand, OLIF restored the ADH, PDH, and DH effectively by the implantation of a larger interbody cage *via* the lateral approach. This result is compatible with previous studies. Sato et al. confirmed that OLIF can significantly improve the DH and spinal canal area (28). The clinical symptoms were relieved by reducing the bulging disc and stretching the redundant ligamentum flavum. Furthermore, the improvement ratio of these parameters is greater in the Non-PD group than in the PD group. The reason may be that the collapse of intervertebral discs was more severe in the Non-PD group preoperatively. More potential restoration of DH is expected. Surprisingly, the FH was increased only in the Non-PD group postoperatively. Chang et al. stated that OLIF showed favorable outcomes in the restoration of FH and that the improvement ratio of the FH was correlated with radicular pain and disability (29). This may explain why patients in the Non-PD group had better clinical outcomes than those in the PD group.

There is no significant improvement in LL and SL after OLIF in this study. Previous literature stated that LLIF had great capacity for coronal deformity correction, but the ability to achieve sagittal plane correction is limited (30). Recently, some studies showed marked sagittal deformity correction in OLIF (31, 32). More studies are needed to discuss the change in sagittal parameters in OLIF.

There are some debates on the indirect decompression effect of the lateral interbody fusion technique. Wang et al. described evidence that bony lateral recess stenosis is an independent risk factor for the failure of the indirect decompression in XLIF (33). Oliveira et al. concluded that congenital stenosis or locked facets may limit the efficacy of indirect decompression in XLIF (10). XLIF is relatively contraindicated for severe central spinal stenosis due to a risk of the need for secondary operation. In addition, a previous study suggested open laminectomy in the presence of fused facet joints or large herniated discs (34). However, some studies had the opposite opinion about these points. Malham et al. and Park et al. reported that facet degeneration does not impair the amount of direct decompression in XLIF (25, 35). Another study announced that locked facets are not a relative contraindication for XLIF (36). A recent systematic review found that only severe central canal stenosis in preoperative images is likely to cause failure of indirect decompression in XLIF (22). In contrast to the experience in XLIF, most articles about OLIF excluded these factors (6, 7, 29). According to our reports, the indirect decompression effect *via* OLIF is sufficient for lumbar FS. If the patients' symptoms were mainly caused by neural compression at neuroforamen, OLIF without direct posterior decompression is a reasonable treatment. Additional direct decompression may be not beneficial in these cases, irrespective of whether facet degeneration is present. However, if the patients are diagnosed with severe central canal stenosis, obvious osteophyte compromising lateral recess, or large disc herniation, direct posterior decompression is considered.

OLIF is a relatively safe procedure with few postoperative minor complications. Postoperative ileus occurred in two patients due to a manipulation of the retroperitoneum. The symptoms improved during hospitalization. About 15% of patients experienced numbness of the anterior thigh after operation with intact motor function. The sensory deficit may be caused by a retraction of the genitofemoral nerve (24), and it was relieved spontaneously within three months of follow-up at the outpatient clinic. Dural tear happened in one patient when laminectomy was performed. The tear site was repaired by tissue glue and CSF leakage was checked meticulously. The patient had no associated complication afterward.

This study had some limitations. First, this was a retrospective study. The plan for additional direct posterior decompression depended on the patients' decision and the surgeons' preference. This may have led to a patient selection bias. Second, this was a mid-term follow-up study, with a median follow-up time of 31.69 months. Third, this was a single-center study, and thus, its generalizability may be inadequate. Fourth, the number of patients was limited, and a larger sample size is necessary in further studies.

The use of OLIF for lumbar FS showed favorable clinical and radiologic outcomes during the 2-year follow-up period. Moreover, the use of interbody cages and posterior instrumentation without direct decompression was sufficient for the relief of symptoms in patients with lumbar FS. Additionally, direct posterior decompression may not be necessary in these patients.

## Data Availability

The raw data supporting the conclusions of this article will be made available by the authors, without undue reservation.
